# Health care resource use and costs associated with adult pneumococcal disease in the United States from 2017 to 2019, stratified by age and health risk: a retrospective cohort study

**DOI:** 10.3389/fpubh.2025.1575125

**Published:** 2025-07-15

**Authors:** Nicole Cossrow, M. Doyinsola Bailey, Yi-Ling Huang, Lei Ai, Salini Mohanty, Valina C. McGuinn, Kelly D. Johnson

**Affiliations:** Merck & Co., Inc., Rahway, NJ, United States

**Keywords:** pneumococcal disease, invasive pneumococcal disease, non-bacteremic pneumococcal pneumonia, United States, health care resource use, health economics

## Abstract

**Background:**

Adult pneumococcal disease (PD) represents a significant clinical and economic burden in the United States. Individuals with immunocompromising conditions and other chronic medical conditions, as well as those ≥65 years of age, have an increased risk of acute PD and its long-term complications. The aim of the current study was to describe the health care resource use and direct health care costs associated with invasive PD (IPD) and non-bacteremic pneumococcal pneumonia (NBPP) among adults in the United States, stratified by age group and health-based risk level.

**Methods:**

This was a retrospective study of administrative claims from the Merative™ MarketScan® Commercial Database from 2017 to 2019. The study population comprised individuals ≥18 years of age with ≥1 episode of IPD (with hospitalization) or NBPP (with or without hospitalization) during the study period. The study outcomes were the PD-associated health care resource use (outpatient visits and length of any hospitalizations, in days) and direct health care costs per episode.

**Results:**

The average health care resource use and direct costs associated with PD were significantly higher for IPD (mean [95% CI] overall cost $49,481 [$45,803–53,159] per episode; *N* = 949 affected individuals) than for NBPP with hospitalization ($27,330 [$23,807–30,852] per episode; *N* = 389) and NBPP without hospitalization ($1,090 [$927–1,252] per episode; *N* = 1,951). For IPD and for NBPP without hospitalization, the direct costs of treatment were significantly higher among groups with immunocompromising or other relevant comorbidities. The costs associated with NBPP without hospitalization were also significantly higher in the oldest age group (≥65 years).

**Conclusion:**

Targeting PD prevention efforts to high-risk groups based on age and/or health risk level could decrease the clinical and economic burden of adult PD in the US.

## 1 Introduction

Pneumococcal disease (PD) is caused by the bacterium *Streptococcus pneumoniae* (1, 2). The most common form of adult invasive PD (IPD)—an infection in a sterile anatomical site—is bacteremic pneumococcal pneumonia, while non-invasive forms include non-bacteremic pneumococcal pneumonia (NBPP) (1, 2). Severe PD, and especially severe IPD, is associated with a high risk of hospitalization, death, and complications such as new or exacerbated chronic medical conditions (CMCs) affecting various organs (1–3). Adults ≥65 years of age, and adults of any age with immunocompromising conditions (ICs) or certain other CMCs, are at particularly high risk of acute PD and its long-term complications (4–12).

Effective pneumococcal vaccines are available in the United States (US) and recommended by the Centers for Disease Control and Prevention’s Advisory Committee on Immunization Practices (ACIP) for high-risk adults: i.e., those ≥19 years of age with an IC or CMC, and all older adults (the prior recommendation for adults ≥65 years of age was updated in October 2024 to include all adults ≥50 years of age) (13–17). However, vaccine uptake among these vulnerable sub-populations remains low (14–21) and PD is still responsible for a substantial disease burden among US adults (2, 6, 7, 9–12, 22–27).

Despite this substantial burden, there is limited information on how known age- and health-based risk factors for PD affect health care resource use (HCRU) and costs. The available evidence generally suggests that age- and comorbidity-related risk factors for PD also increase the levels of HCRU and the costs associated with the treatment of PD, but these relationships are not consistent across studies and disease types (7, 9–12, 25, 28). In addition, many of these studies did not distinguish between inpatient and outpatient care, while many studies assessed broad diagnoses such as ‘all-cause pneumonia’ or ‘community-acquired pneumonia’ that are not necessarily specific to pneumococcal forms of the disease.

Comprehensive up-to-date information on the HCRU and costs associated with different forms of adult PD among different age and health risk groups would help inform future disease prevention and treatment efforts. The objectives of the current study were therefore to describe the HCRU and direct health care costs of IPD and NBPP among US adults, overall and stratified by hospitalization status, age, and health-based risk level.

## 2 Materials and methods

### 2.1 Study design and data source

This was a retrospective cohort study of US adults ≥18 years of age who had a diagnosis of IPD or NBPP between January 1, 2017 and December 31, 2019. This study period reflects the most recent available data from before the COVID-19 pandemic; the public health measures enacted during the first phases of the pandemic affected the incidence of PD and may also have affected the delivery and costs of health care for PD. Data were obtained from the Merative™ MarketScan® Commercial Database, which contains de-identified administrative data on enrollee sociodemographic characteristics, diagnoses, prescriptions, HCRU, and associated direct health care costs in inpatient, outpatient, and pharmacy settings. As no identifiable data were included in the study, no specific informed consent or ethics committee approval was required.

### 2.2 Study population

Claims from individuals who were enrolled in a commercial health insurance plan included in the study database during the study period (January 1, 2015–December 31, 2019) and had ≥1 diagnosis of PD between January 1, 2017 and December 31, 2019 were eligible for inclusion. The index date was the first date of diagnosis with PD within each study year (2017, 2018, or 2019). Pneumococcal disease was identified using International Classification of Diseases, Tenth Revision (ICD-10) codes with clinical modification in inpatient and/or outpatient claims (Supplementary Table 1). Eligible PD episodes were associated with a specific diagnosis of IPD (with hospitalization) or NBPP (with or without hospitalization) on the index date. A single episode was defined as including all uses of any PD code within a 90-day period beginning on the index date. Hospitalization was identified by any evidence of an inpatient claim with IPD or NBPP as the primary diagnosis within a calendar year following the index date. Episodes with a diagnosis of IPD but no record of hospitalization were excluded, as IPD is generally a very serious condition that requires hospitalization, and outpatient-only episodes were likely associated with misdiagnoses or miscoded claims. We also excluded episodes of recurrent PD, defined as a diagnosis of any form of PD within 30 days prior to the index date. Additional eligibility criteria were enrollee age of ≥18 years on the index date, with continuous enrollment (i.e., no insurance coverage gaps of >45 days) for ≥2 years before and ≥6 months after the index date.

Each individual was assigned to one or more annual cohorts based on the calendar year(s) of their index date(s), as well as the relevant age group (18–49, 50–64, or ≥65 years of age on index date) and health risk category. Health risk was defined per ACIP’s current pneumococcal vaccination recommendations (Supplementary Table 2) and assigned based on evidence for the use of relevant ICD, National Drug Code, and/or Healthcare Common Procedure Coding System/Current Procedural Terminology (HCPCS/CPT-4) codes (available upon request) during the 2-year period immediately preceding the index date (15, 29). Individuals with the highest risk were those with ≥1 IC, cochlear implant, or cerebrospinal fluid leak (‘IC’ group), while the moderate risk group comprised individuals with no IC but ≥1 relevant CMC (‘CMC’ group). Individuals with ≥2 relevant conditions from different risk categories were assigned to the higher-risk category. All other individuals were assigned to the low-risk ‘Healthy’ group.

### 2.3 Variables

Health care resource use associated with PD was defined as any inpatient or outpatient encounter that was associated with an individual’s first eligible episode of PD in a given calendar year. The total number of PD-related outpatient visits, and the length in days of any PD-related hospitalizations, were recorded for each individual. The unadjusted costs of all instances of PD-related HCRU were also extracted from the database and classified as inpatient, outpatient, or emergency department (ED) costs per episode.

### 2.4 Statistical analysis

The PD-related HCRU and health care costs per episode were analyzed using descriptive statistics (mean, standard deviation [SD], and 95% confidence interval [CI]). Analyses were stratified by annual cohort, diagnosis and associated hospitalization status, and age and/or health risk group.

## 3 Results

### 3.1 Pneumococcal disease incidence stratified by annual cohort, age group, and health risk group

The annual number of individuals with ≥1 episode of each PD decreased during the study period: in 2017 there were 342 individuals with ≥1 diagnosis of IPD, 159 of NBPP with hospitalization, and 880 of NBPP with no hospitalization, compared to 285, 117, and 490 individuals, respectively, in 2019 (Table 1). Most affected individuals were in the 2 oldest age groups and/or 2 highest health risk groups. For example, a combined 79.3% of all individuals with ≥1 episode of IPD, 81.7% of those with NBPP with hospitalization, and 73.4% of those with NBPP without hospitalization were in the 50–64 or ≥65 years of age groups, while the combined CMC and IC groups accounted for 91.6% of all individuals with a diagnosis of IPD, 92.7% of those with NBPP with hospitalization, and 80.2% of those with NBPP without hospitalization.

**Table 1:** Number of individuals with ≥1 episode of pneumococcal disease, stratified by annual cohort, age, and risk group.

Group	IPD			NBPP (hospitalized)			NBPP (non-hospitalized)		
	2017	2018	2019	2017	2018	2019	2017	2018	2019
Overall	342	322	285	159	123	117	880	581	490
Age (years)									
18–49	64	64	68	26	26	21	214	162	142
50–64	157	166	134	63	48	59	337	252	226
≥65	121	92	83	70	49	37	329	167	122
Health risk									
Healthy	25	35	20	14	9	6	158	116	113
CMC	146	120	121	72	54	52	461	264	217
IC	171	167	144	73	60	59	261	201	160
Age/health risk									
18–49, Healthy	12	15	8	5	4	4	77	61	55
18–49, CMC	21	21	27	6	12	12	103	71	58
18–49, IC	31	28	33	15	10	5	34	30	29
50–64, Healthy	10	16	11	8	4	0	64	46	52
50–64, CMC	76	59	55	32	20	27	190	119	100
50–64, IC	71	91	68	23	24	32	83	87	74
≥65, Healthy	3	4	1	1	1	2	17	9	6
≥65, CMC	49	40	39	34	22	13	168	74	59
≥65, IC	69	48	43	35	26	22	144	84	57

CMC, chronic medical condition; IC, immunocompromising condition; IPD, invasive pneumococcal disease; NBPP, non-bacteremic pneumococcal pneumonia.

All individuals with invasive pneumococcal disease were hospitalized.

### 3.2 Invasive pneumococcal disease

Among the 3 annual cohorts, IPD was associated with an overall average of 5.3–5.7 outpatient visits per episode and a hospital stay of 8.8–10.3 days (Table 2). Hospitalizations were generally longer for older individuals, with a range among the 3 annual cohorts of 9.8–11.1 days for those 50–64 years of age and 7.7–10.3 days for those ≥65 years of age, compared to 7.9–8.6 days for those 19–49 years of age. Individuals with a higher health risk status also had longer hospitalizations than those in the Healthy group (8.9–12.8 days for the CMC group and 8.4–9.4 days for the IC group, compared to 6.3–8.3 days for the Healthy group). This pattern was consistent across all age groups.

**Table 2:** Per episode health care resource use associated with invasive pneumococcal disease, stratified by annual cohort, age, and risk group.

Group	Length of hospital stay (days)			Number of outpatient visits		
	2017	2018	2019	2017	2018	2019
Overall	9.2 (8.1)	8.8 (7.4)	10.3 (14.0)	5.3 (8.2)	5.3 (8.1)	5.7 (7.3)
Age (years)						
18–49	8.2 (5.4)	7.9 (6.7)	8.6 (7.5)	5.5 (8.9)	4.5 (4.8)	4.5 (5.1)
50–64	9.8 (9.2)	9.8 (8.5)	11.1 (17.5)	5.4 (8.1)	6.1 (8.8)	6.5 (8.6)
≥65	9.0 (7.7)	7.7 (4.9)	10.3 (11.8)	5.0 (7.8)	4.5 (8.6)	5.3 (6.4)
Health risk						
Healthy	7.8 (4.8)	6.3 (4.2)	8.3 (8.4)	2.5 (2.5)	5.7 (6.1)	4.5 (4.3)
CMC	9.3 (8.0)	8.9 (7.7)	12.8 (19.9)	5.4 (8.8)	4.5 (7.9)	4.9 (6.2)
IC	9.4 (8.5)	9.3 (7.6)	8.4 (6.4)	5.6 (8.1)	5.9 (8.6)	6.5 (8.3)

CMC, chronic medical condition; IC, immunocompromising condition.

All values are represented as the mean (standard deviation). All individuals with invasive pneumococcal disease were hospitalized.

**Figure 1:**
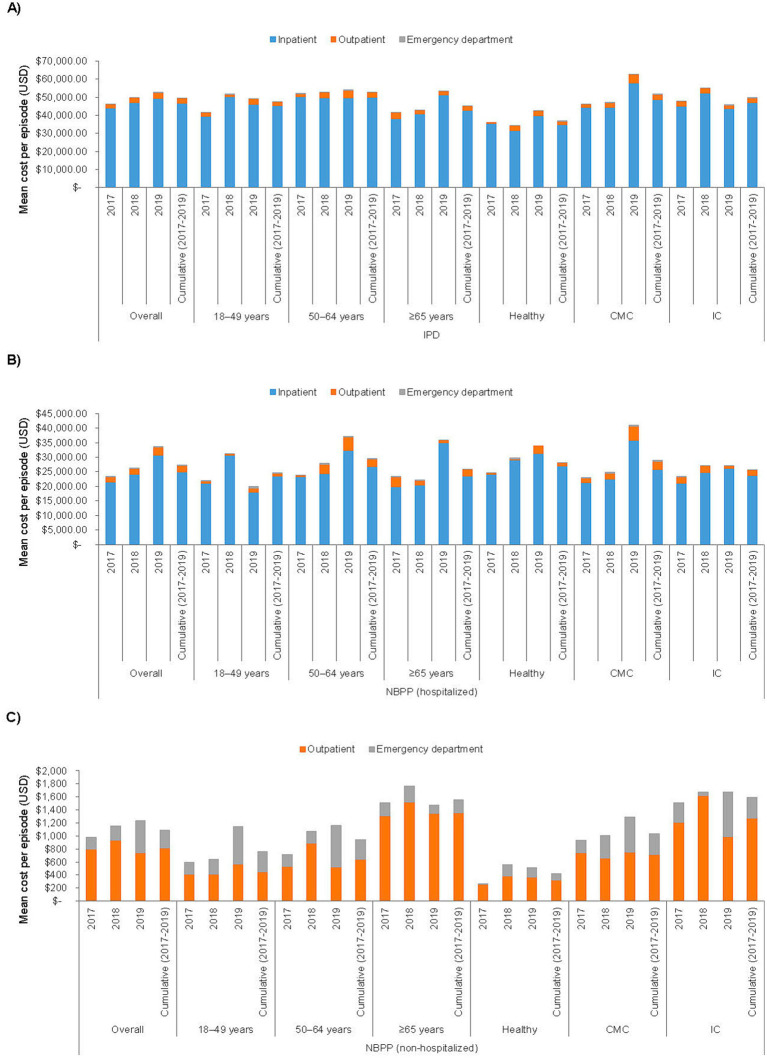
Cost per episode of **(A)** invasive pneumococcal disease, **(B)** non-bacteremic pneumococcal pneumonia with hospitalization, and **(C)** non-bacteremic pneumococcal pneumonia with no hospitalization, stratified by annual cohort, age, and risk group. CMC, chronic medical condition; IC, immunocompromising condition; IPD, invasive pneumococcal disease; NBPP, non-bacteremic pneumococcal pneumonia; USD, United States Dollar.

The overall average cost per episode of pneumococcal disease across all 3 annual cohorts are summarized in Table 3, stratified by age and/or health risk group; detailed breakdowns by annual cohort and cost type (inpatient, outpatient, and ED) are presented in Figure 1A and Supplementary Table 3. Inpatient costs contributed the bulk of the overall costs ($43,888–49,266 per episode in the overall annual cohort groups, compared to $2,401–3,359 for outpatient and $88–117 for ED costs).

**Table 3:** Total cost per episode of pneumococcal disease 2017–2019, stratified by age and risk group.

Group	IPD		NBPP (hospitalized)		NBPP (non-hospitalized)	
	Mean (SD)	95% CI	Mean (SD)	95% CI	Mean (SD)	95% CI
Overall	49,481 (57,734)	45,803, 53,159	27,330 (35,786)	23,807, 30,852	1,090 (3,672)	927, 1,252
Age (years)						
18–49	47,606 (56,004)	39,717, 55,496	24,711 (21,591)	19,674, 29,749	756 (2,658)	527, 985
50–64	52,958 (60,258)	47,419, 58,498	29,678 (42,804)	23,198, 36,159	945 (3,774)	686, 1,204
≥65	45,353 (54,656)	39,101, 51,605	25,995 (32,605)	20,838, 31,152	1,559 (4,190)	1,230, 1,888
Health risk						
Healthy	36,951 (31,519)	29,936, 43,965	28,213 (29,186)	17,112, 39,315	423 (1,157)	308, 538
CMC	51,685 (59,741)	45,714, 57,655	28,879 (44,141)	22,350, 35,409	1,029 (3,235)	823, 1,235
IC	49,791 (59,262)	44,487, 55,095	25,759 (27,079)	21,905, 29,614	1,598 (5,014)	1,204, 1,992
Age/health risk						
18–49, Healthy	43,215 (36,469)	30,687, 55,742	30,523 (29,750)	12,545, 48,501	380 (1,063)	229, 531
18–49, CMC	50,104 (54,739)	36,954, 63,254	23,666 (21,385)	15,681, 31,651	936 (3,543)	481, 1,392
18–49, IC	47,404 (63,029)	34,351, 60,457	23,238 (17,759)	16,607, 29,870	1,080 (2,276)	611, 1,548
50–64, Healthy	33,993 (28,264)	24,570, 43,417	18,532 (19,365)	6,229, 30,836	453 (1,276)	257, 649
50–64, CMC	56,387 (67,500)	46,727, 66,047	33,263 (57,923)	20,289, 46,237	757 (2,244)	539, 975
50–64, IC	53,177 (57,241)	45,740, 60,614	27,787 (22,938)	22,649, 32,925	1,592 (6,139)	818, 2,367
≥65, Healthy	23,221 (13,623)	11,832, 34,610	49,749 (44,929)	21,743, 121,240	530 (1,082)	140, 920
≥65, CMC	45,556 (48,890)	37,005, 54,107	26,127 (31,404)	18,583, 33,672	1,468 (3,993)	1,017, 1,918
≥65, IC	46,297 (59,996)	36,929, 55,665	24,740 (32,995)	17,536, 31,945	1,770 (4,580)	1,239, 2,301

CI, confidence interval; CMC, chronic medical condition; IC, immunocompromising condition; IPD, invasive pneumococcal pneumonia; NBPP, non-bacteremic pneumococcal pneumonia; SD, standard deviation.

All values are expressed in US dollars and represent average total (inpatient, outpatient, and emergency department) costs across all 3 annual cohorts (2017, 2018, and 2019). All individuals with invasive pneumococcal disease were hospitalized. Detailed breakdowns by annual cohort and type of cost are available in Supplementary Tables 3–5.

The overall per episode cost of IPD was similar across all 3 age groups, overall and within each health risk category. Costs were also similar between the CMC and the IC health risk groups, but each of these groups incurred significantly higher costs than the Healthy group (Healthy, mean [SD] $36,951 [$31,519] per episode, 95% CI $29,936–43,965; CMC, $51,685 [$59,741], $45,714–57,655; IC, $49,791 [$59,262], $44,487–55,095). The relationship between health risk and costs differed by age group: for individuals 18–49 years of age, costs were similar across all 3 health risk subgroups, whereas within the 50–64 and the ≥65 years of age groups, both the CMC and the IC subgroups had significantly higher costs compared to the respective Healthy subgroup.

### 3.3 Non-bacteremic pneumococcal pneumonia

Compared to IPD, NBPP with hospitalization was associated with fewer outpatient visits (mean 3.4–4.0 per episode among the 3 overall annual cohorts) and shorter hospital stays (6.3–6.5 days; Table 4). NBPP with no hospitalization was associated with even lower HCRU (2.7–2.9 outpatient visits per episode among the overall annual cohorts). The cost of NBPP with hospitalization was significantly lower than that of IPD when assessed overall (NBPP, mean [SD] $27,330 [$35,786] per episode, 95% CI $23,807–30,852 versus IPD, $49,481 [$57,734], $45,803–53,159), among all 3 age groups, and among the CMC and IC health risk groups, with and without further stratification by age group (Table 3; Figure 1B; Supplementary Table 4). The average cost of NBPP with no hospitalization was significantly lower than that of both IPD and NBPP with hospitalization, overall (mean [SD] $1,090 [$3,672] per episode, 95% CI $927–1,252) and across all age and/or health risk groups and subgroups (Table 3; Figure 1C; Supplementary Table 5).

**Table 4:** Per episode health care resource use associated with non-bacteremic pneumococcal pneumonia with hospitalization, stratified by annual cohort, age, and risk group.

Group	Hospitalized individuals						Non-hospitalized individuals		
	Length of hospital stay (days)			Number of outpatient visits			Number of outpatient visits		
	2017	2018	2019	2017	2018	2019	2017	2018	2019
Overall	6.4 (4.8)	6.3 (4.8)	6.5 (4.5)	3.7 (6.0)	3.4 (5.4)	4.0 (5.6)	2.7 (4.6)	2.7 (3.8)	2.9 (4.3)
Age (years)									
18–49	5.5 (2.4)	7.4 (7.2)	4.7 (2.2)	3.7 (4.7)	3.8 (5.7)	5.0 (5.9)	1.8 (2.6)	2.3 (3.6)	2.2 (3.3)
50–64	6.1 (4.1)	6.5 (4.6)	6.9 (5.3)	2.6 (3.3)	4.1 (7.1)	3.9 (5.8)	2.6 (5.1)	2.8 (3.7)	2.6 (3.4)
≥65	6.9 (6.0)	5.6 (3.3)	6.8 (3.7)	4.8 (8.0)	2.3 (2.6)	3.6 (5.3)	3.5 (5.0)	3.1 (4.0)	4.2 (6.1)
Health risk									
Healthy	5.4 (2.7)	6.9 (7.2)	5.0 (2.6)	2.8 (3.2)	2.7 (2.4)	4.5 (4.9)	1.5 (1.0)	1.9 (3.4)	1.9 (1.9)
CMC	6.4 (3.8)	6.0 (5.2)	6.8 (5.2)	2.9 (3.3)	3.2 (6.2)	4.2 (5.9)	2.7 (4.9)	2.5 (2.7)	2.8 (4.4)
IC	6.6 (6.0)	6.5 (4.1)	6.4 (4.0)	4.7 (8.1)	3.6 (5.0)	3.9 (5.6)	3.4 (5.3)	3.5 (4.8)	3.7 (5.1)

CMC, chronic medical condition; IC, immunocompromising condition.

All values are represented as the mean (standard deviation).

There was no statistically significant association between health risk or age group and the cost of NBPP with hospitalization (Table 3). In contrast, for NBPP with no hospitalization, costs were significantly higher among those ≥65 years of age compared to those 50–64 or 18–49 years of age, as well as among the CMC and IC groups compared to the Healthy group.

## 4 Discussion

In this study, we determined that the HCRU associated with an episode of IPD or NBPP was generally higher among older adults and among individuals with ≥1 IC or ≥1 CMC. The costs of IPD and of NBPP without hospitalization were significantly positively associated with health risk level, and the costs of NBPP without hospitalization were also significantly associated with age.

Across all age and risk groups and annual cohorts, the mean hospitalization length ranged from 4.7 to 7.4 days per episode for NBPP and from 6.3 to 12.8 days for IPD. These findings are generally consistent with the existing US literature. For instance, previous analyses reported average hospital stays per episode of IPD of 11.2 days (among adults ≥18 years of age), 9.7–12.2 days (≥18 years), or 6.1–8.4 days (19–64 years with ≥1 CMC or IC) (10, 12, 30). Studies of pneumonia-related HCRU have reported mean hospital stays of 4.6–5.6 days per episode of inpatient pneumonia (≥18 years), and a mean hospitalization time of 6.0 days for NBPP (19–64 years with ≥1 CMC or IC) or 1.4–4.0 days for all-cause pneumonia (19–64 years with ≥1 CMC or IC) (7, 12, 30). In contrast, another study reported longer average hospitalizations (11.0–23.4 days) for all-cause pneumonia among adults ≥18 years of age (10).

We also found that IPD was associated with a mean of 2.5–6.5 outpatient visits per episode, compared to 2.3–5.0 visits for NBPP with hospitalization and 1.6–4.2 visits for NBPP with no hospitalization. These values were generally consistent with previous studies, which observed ranges of 1.8–4.0 (≥18 years) or 0.1–0.2 (19–64 years with ≥1 CMC or IC) visits per episode of IPD (10, 12), 1.2–1.8 visits per episode of inpatient pneumonia or 1.0–1.1 visits per episode of outpatient pneumonia (≥18 years) (7), and 1.4–2.3 (≥18 years) or 0.2–0.6 (19–64 years with ≥1 CMC or IC) visits per episode of all-cause pneumonia (10, 12).

Our estimated mean costs across all age and/or health risk groups were $23,221–56,387 per episode for IPD, $18,532–49,749 for NBPP with hospitalization, and $380–1,770 for NBPP with no hospitalization. Previous analyses using the same database reported generally similar ranges of $10,723–71,198 per episode of IPD (19–64 years with ≥1 CMC or IC) (12), and $2,178–4,993 (≥18 years) or $4,725–11,847 (19–64 years with ≥1 CMC or IC) per episode of all-cause pneumonia (9, 12). A study that used the same database in combination with 2 others estimated overall per episode costs of $20,897–43,349 for IPD and $4,247–16,775 for pneumonia (≥18 years) (10).

The costs associated with IPD and NBPP without hospitalization were significantly higher among the IC and/or CMC groups compared with healthy individuals; the cost of NBPP with no hospitalization was also significantly higher among the oldest age group than among younger adults. Several prior pneumonia studies identified similar associations (7, 9–12, 25, 28). For example, a claims database study found that the mean length of hospitalization for all-cause pneumonia was 1.4 days for healthy individuals, 2.4 days for individuals with ≥1 CMC, and 3.5 days for those with ≥1 IC (12). In a previous database study of the impact of age on PD-related costs, the mean cost per episode of pneumonia was estimated to range from $2,178 among adults 18–49 years of age to $4,993 among those ≥85 years of age (9, 25). In a similar analysis based on health risk rather than age group, the estimated mean cost per episode of pneumonia was $4,725 among healthy adults, $6,534 among those with ≥1 CMC, and $9,168 among those with ≥1 IC (12).

Fewer studies have assessed the HCRU and costs associated with IPD. Two previous claims database studies identified a clear positive association between both age and risk level and the HCRU associated with pneumonia, but not with IPD (10, 12). One of these analyses noted that the results for IPD were ‘not robust due to the relatively small number of identified episodes’, and also noted that the reduced prevalence of ICs among older age groups may have affected the results (10); a similar observation was included in the other study (12). While one of these studies found that the costs per episode of IPD were similar across all health risk levels (12), the other found that the younger of the 2 age groups assessed (18–64 years of age) incurred significantly higher costs per episode of IPD than did the older age group (≥65 years of age) (10). The current analysis stratified the study population into 3 age groups rather than 2 and found that the numerically highest costs of IPD were generally incurred by individuals 50–64 years of age, which may be consistent with the previous study (10). In addition, the database used in both analyses may not capture all claims for individuals ≥65 years of age with partial Medicare coverage, and thus the estimated HCRU and costs for this oldest age group may have been underestimated in both studies (10, 31).

The aging US population, as well as the increasing prevalence of relevant CMCs and the spread of antimicrobial-resistant strains of *S. pneumoniae*, are predicted to increase the clinical and economic burden of PD in the US in the coming years (26, 32–34). These threats necessitate robust surveillance programs to monitor the spread of antimicrobial resistance and its impact on the economic burden of PD, as well as improved and more equitable preventive measures. Pneumococcal vaccination has consistently proven to be a cost-effective intervention in the US that reduces PD-associated HCRU and direct and indirect costs, especially among individuals with age- or comorbidity-related risk factors for PD (35–39). As well as preventing PD, there is some evidence that adult pneumococcal vaccination can also improve clinical outcomes and reduce the HCRU and costs associated with breakthrough infections compared to similar episodes of PD among unvaccinated adults (40–44). Increasing the uptake of adult pneumococcal vaccination may thus be a cost-effective means to reduce the overall burden of PD and decrease racial disparities in PD outcomes in the US (45, 46).

The large size of our study population allowed us to rigorously assess the HCRU and costs associated with different specific PD diagnoses, stratified by hospitalization status, age group, and health risk level. These data are particularly valuable for IPD, for which small sample sizes have hindered previous attempts to identify factors that affect these outcomes.

The study is also subject to some known limitations. As with any claims database, diagnostic miscoding may occur, potentially resulting in misclassification and measurement errors, which may have affected the accuracy of our estimates. Our cost estimates comprise only the direct insurer-paid costs of medical care and do not reflect the true economic burden of PD (35). However, the direct costs of PD-associated health care, especially inpatient care, are the primary driver of the overall economic burden of adult PD (7, 9–12, 23–27), and our data thus capture a substantial portion of the overall economic burden of disease. Further, although the study database is considered representative of US individuals with commercial health insurance obtained through large employers, it is not representative of the entire adult US population (31). The lack of representation of adults without insurance (an estimated 8.0% of Americans in 2019 (47)), those with commercial insurance through smaller employers, those covered under Medicaid (an estimated 18.1% of Americans in 2019 (47)), or adults ≥65 years of age who obtain their insurance exclusively through Medicare may have introduced sociodemographic, clinical, or other biases that may have affected the study’s findings. For example, adults with Medicaid or Medicare insurance are likely to be older and have a higher prevalence of ICs and CMCs than commercially insured adults, which would be expected to affect both the incidence and outcomes of PD (48, 49). As a result, we likely underestimated the true incidence and cost of PD among the oldest and most medically fragile groups. The Merative™ MarketScan® Commercial Database does not include individual-level socioeconomic data, such as income or race/ethnicity. Consequently, differences in the incidence of PD risk factors and outcomes, including HCRU and costs, across racial/ethnic and socioeconomic groups may have affected the generalizability of the study (50, 51).

In conclusion, PD is associated with substantial HCRU and health care costs among US adults, particularly those with ICs or CMCs. Preventing PD via pneumococcal vaccination offers a means to reduce this clinical and economic burden of disease.

## Data Availability

The original contributions presented in the study are included in the article/Supplementary material, further inquiries can be directed to the corresponding author.

## References

[1] Drijkoningen JJ Rohde GG. Pneumococcal infection in adults: burden of disease. Clin Microbiol Infect. (2014) 20:45–51. doi: 10.1111/1469-0691.12461 24313448

[2] Gierke R McGee L Beall B Pilishivili T. Pneumococcal disease In: Roush SW Baldy LM, editors. Manual for the surveillance of vaccine-preventable diseases: Centers for Disease Control and Prevention (2020)

[3] Kruckow KL Zhao K Bowdish DME Orihuela CJ. Acute organ injury and long-term sequelae of severe pneumococcal infections. Pneumonia (Nathan). (2023) 15:5. doi: 10.1186/s41479-023-00110-y, PMID: 36870980 PMC9985869

[4] Centers for Disease Control and Prevention. Epidemiology and prevention of vaccine-preventable diseases. (2016). Available online at: https://www.cdc.gov/vaccines/pubs/pinkbook/chapters.html (Accessed April 3, 2019).

[5] Davidson RN Wall RA. Prevention and management of infections in patients without a spleen. Clin Microbiol Infect. (2001) 7:657–60. doi: 10.1046/j.1198-743x.2001.00355.x, PMID: 11843905

[6] Grant LR Meche A McGrath L Miles A Alfred T Yan Q. Risk of pneumococcal disease in US adults by age and risk profile. Open Forum Infect Dis. (2023) 10:–ofad192. doi: 10.1093/ofid/ofad192, PMID: 37180598 PMC10167987

[7] Huang SS Johnson KM Ray GT Wroe P Lieu TA Moore MR. Healthcare utilization and cost of pneumococcal disease in the United States. Vaccine. (2011) 29:3398–412. doi: 10.1016/j.vaccine.2011.02.088, PMID: 21397721

[8] Kuchar E Miskiewicz K Karlikowska M. A review of guidance on immunization in persons with defective or deficient splenic function. Br J Haematol. (2015) 171:683–94. doi: 10.1111/bjh.13660, PMID: 26315210

[9] Tong S Amand C Kieffer A Kyaw MH. Trends in healthcare utilization and costs associated with pneumonia in the United States during 2008-2014. BMC Health Serv Res. (2018) 18:715. doi: 10.1186/s12913-018-3529-4 30217156 PMC6137867

[10] Weycker D Farkouh RA Strutton DR Edelsberg J Shea KM Pelton SI. Rates and costs of invasive pneumococcal disease and pneumonia in persons with underlying medical conditions. BMC Health Serv Res. (2016) 16:182. doi: 10.1186/s12913-016-1432-4 27177430 PMC4867996

[11] Weycker D Strutton D Edelsberg J Sato R Jackson LA. Clinical and economic burden of pneumococcal disease in older US adults. Vaccine. (2010) 28:4955–60. doi: 10.1016/j.vaccine.2010.05.030, PMID: 20576535

[12] Zhang D Petigara T Yang X. Clinical and economic burden of pneumococcal disease in US adults aged 19-64 years with chronic or immunocompromising diseases: an observational database study. BMC Infect Dis. (2018) 18:436. doi: 10.1186/s12879-018-3326-z 30157781 PMC6116536

[13] Centers for Disease Control and Prevention. CDC recommends lowering the age for pneumococcal vaccination from 65 to 50 years old. (2024). Available online at: https://www.cdc.gov/media/releases/2024/s1023-pneumococcal-vaccination.html (Accessed December 5, 2024).

[14] Kobayashi M Leidner AJ Gierke R Farrar JL Morgan RL Campos-Outcalt D. Use of 21-valent pneumococcal conjugate vaccine among U.S. adults: recommendations of the advisory committee on immunization practices - United States, 2024. MMWR Morb Mortal Wkly Rep. (2024) 73:793–8. doi: 10.15585/mmwr.mm7336a3, PMID: 39264843 PMC11392227

[15] Kobayashi M Pilishvili T Farrar JL Leidner AJ Gierke R Prasad N. Pneumococcal vaccine for adults aged ≥19 years: recommendations of the advisory committee on immunization practices, United States, 2023. MMWR Recomm Rep. (2023) 72:1–39. doi: 10.15585/mmwr.rr7203a1, PMID: 37669242 PMC10495181

[16] Musher DM Anderson R Feldman C. The remarkable history of pneumococcal vaccination: an ongoing challenge. Pneumonia (Nathan). (2022) 14:5. doi: 10.1186/s41479-022-00097-y 36153636 PMC9509586

[17] US Food & Drug Administration. CAPVAXIVE. (2024). Available online at: https://www.fda.gov/vaccines-blood-biologics/capvaxive (Accessed July 23, 2024).

[18] Centers for Disease Control and Prevention. Vaccination coverage among adults in the United States, National Health Interview Survey. (2021). Available online at: https://www.cdc.gov/vaccines/imz-managers/coverage/adultvaxview/pubs-resources/vaccination-coverage-adults-2021.html#:~:text=Coverage%20with%20at%20least%20one,%E2%80%93September%202021%20(17) (Accessed December 7, 2022).

[19] Williams WW Lu PJ O'Halloran A Kim DK Grohskopf LA Pilishvili T. Surveillance of vaccination coverage among adult populations - United States, 2015. MMWR Surveill Summ. (2017) 66:1–28. doi: 10.15585/mmwr.ss6611a1, PMID: 28472027 PMC5829683

[20] Yang X Zhang D Ou W. Pneumococcal vaccination patterns among persons aged 65 years or older in the United States: a retrospective database analysis. Vaccine. (2018) 36:7574–9. doi: 10.1016/j.vaccine.2018.10.015, PMID: 30391053

[21] Oliveira GS Oliveira MLS Miyaji EN Rodrigues TC. Pneumococcal vaccines: past findings, present work, and future strategies. Vaccines (Basel). (2021) 9:1338. doi: 10.3390/vaccines9111338, PMID: 34835269 PMC8620834

[22] Cossrow N Bailey MD Huang YL Ai L Mohanty S McGuinn VC. Incidence of pneumococcal disease and pneumococcal vaccine uptake among US adults by age and risk groups. 10.1080/03007995.2026.2661502 42043954

[23] De Graeve D Beutels P. Economic aspects of pneumococcal pneumonia: a review of the literature. PharmacoEconomics. (2004) 22:719–40. doi: 10.2165/00019053-200422110-00003, PMID: 15250750

[24] McLaughlin JM McGinnis JJ Tan L Mercatante A Fortuna J. Estimated human and economic burden of four major adult vaccine-preventable diseases in the United States, 2013. J Prim Prev. (2015) 36:259–73. doi: 10.1007/s10935-015-0394-3, PMID: 26032932 PMC4486398

[25] Ozawa S Portnoy A Getaneh H Clark S Knoll M Bishai D. Modeling the economic burden of adult vaccine-preventable diseases in the United States. Health Aff (Millwood). (2016) 35:2124–32. doi: 10.1377/hlthaff.2016.0462, PMID: 27733424

[26] Reynolds CA Finkelstein JA Ray GT Moore MR Huang SS. Attributable healthcare utilization and cost of pneumonia due to drug-resistant streptococcus pneumonia: a cost analysis. Antimicrob Resist Infect Control. (2014) 3:16. doi: 10.1186/2047-2994-3-16 24851182 PMC4029811

[27] Shiri T Khan K Keaney K Mukherjee G McCarthy ND Petrou S. Pneumococcal disease: a systematic review of health utilities, resource use, costs, and economic evaluations of interventions. Value Health. (2019) 22:1329–44. doi: 10.1016/j.jval.2019.06.011, PMID: 31708071

[28] Niederman MS McCombs JS Unger AN Kumar A Popovian R. The cost of treating community-acquired pneumonia. Clin Ther. (1998) 20:820–37. doi: 10.1016/S0149-2918(98)80144-6, PMID: 9737840

[29] Centers for Disease Control and Prevention. Use of 13-valent pneumococcal conjugate vaccine and 23-valent pneumococcal polysaccharide vaccine for adults with immunocompromising conditions: recommendations of the advisory committee on immunization practices (ACIP). MMWR Morb Mortal Wkly Rep. (2012) 61:816–9. PMID: 23051612

[30] Stack SJ Martin DR Plouffe JF. An emergency department-based pneumococcal vaccination program could save money and lives. Ann Emerg Med. (1999) 33:299–303. doi: 10.1016/S0196-0644(99)70366-5, PMID: 10036344

[31] Kulaylat AS Schaefer EW Messaris E Hollenbeak CS. Truven health analytics MarketScan databases for clinical research in colon and rectal surgery. Clin Colon Rectal Surg. (2019) 32:54–60. doi: 10.1055/s-0038-1673354, PMID: 30647546 PMC6327721

[32] Boersma P Black LI Ward BW. Prevalence of multiple chronic conditions among US adults, 2018. Prev Chronic Dis. (2020) 17:E106. doi: 10.5888/pcd17.200130, PMID: 32945769 PMC7553211

[33] Talbird SE La EM Carrico J Poston S Poirrier JE DeMartino JK. Impact of population aging on the burden of vaccine-preventable diseases among older adults in the United States. Hum Vaccin Immunother. (2021) 17:332–43. doi: 10.1080/21645515.2020.1780847, PMID: 32758069 PMC7899694

[34] Wroe PC Finkelstein JA Ray GT Linder JA Johnson KM Rifas-Shiman S. Aging population and future burden of pneumococcal pneumonia in the United States. J Infect Dis. (2012) 205:1589–92. doi: 10.1093/infdis/jis240, PMID: 22448012

[35] Cafiero-Fonseca ET Stawasz A Johnson ST Sato R Bloom DE. The full benefits of adult pneumococcal vaccination: a systematic review. PLoS One. (2017) 12:e0186903. doi: 10.1371/journal.pone.0186903, PMID: 29088258 PMC5663403

[36] Carrico J Talbird SE La EM Poston S Poirrier JE DeMartino JK. Cost-benefit analysis of vaccination against four preventable diseases in older adults: impact of an aging population. Vaccine. (2021) 39:5187–97. doi: 10.1016/j.vaccine.2021.07.029, PMID: 34334253

[37] Chen J O'Brien MA Yang HK Grabenstein JD Dasbach EJ. Cost-effectiveness of pneumococcal vaccines for adults in the United States. Adv Ther. (2014) 31:392–409. doi: 10.1007/s12325-014-0115-y, PMID: 24718851 PMC4003344

[38] Ishigami J Padula WV Grams ME Chang AR Jaar B Gansevoort RT. Cost-effectiveness of pneumococcal vaccination among patients with CKD in the United States. Am J Kidney Dis. (2019) 74:23–35. doi: 10.1053/j.ajkd.2019.01.025, PMID: 30898360

[39] Wateska AR Nowalk MP Lin CJ Harrison LH Schaffner W Zimmerman RK. Pneumococcal vaccination in adults aged >/=65 years: cost-effectiveness and health impact in U.S. populations. Am J Prev Med. (2020) 58:487–95. doi: 10.1016/j.amepre.2019.10.022, PMID: 32001052 PMC7089827

[40] Fisman DN Abrutyn E Spaude KA Kim A Kirchner C Daley J. Prior pneumococcal vaccination is associated with reduced death, complications, and length of stay among hospitalized adults with community-acquired pneumonia. Clin Infect Dis. (2006) 42:1093–101. doi: 10.1086/501354, PMID: 16575726

[41] Imaz A Falco V Penaranda M Jordano Q Martinez X Nadal C. Impact of prior pneumococcal vaccination on clinical outcomes in HIV-infected adult patients hospitalized with invasive pneumococcal disease. HIV Med. (2009) 10:356–63. doi: 10.1111/j.1468-1293.2009.00695.x, PMID: 19490180

[42] Li C Gubbins PO Chen GJ. Prior pneumococcal and influenza vaccinations and in-hospital outcomes for community-acquired pneumonia in elderly veterans. J Hosp Med. (2015) 10:287–93. doi: 10.1002/jhm.2328, PMID: 25676363

[43] Manzur A Izquierdo C Ruiz L Sousa D Bayas JM Celorrio JM. Influence of prior pneumococcal and influenza vaccination on outcomes of older adults with community-acquired pneumonia. J Am Geriatr Soc. (2011) 59:1711–6. doi: 10.1111/j.1532-5415.2011.03541.x, PMID: 21806565

[44] Mykietiuk A Carratala J Dominguez A Manzur A Fernandez-Sabe N Dorca J. Effect of prior pneumococcal vaccination on clinical outcome of hospitalized adults with community-acquired pneumococcal pneumonia. Eur J Clin Microbiol Infect Dis. (2006) 25:457–62. doi: 10.1007/s10096-006-0161-8, PMID: 16773389

[45] Altawalbeh SM Wateska AR Nowalk MP Lin CJ Harrison LH Schaffner W. Pneumococcal vaccination strategies in 50-year-olds to decrease racial disparities: a US societal perspective cost-effectiveness analysis. Value Health. (2024) 27:721–9. doi: 10.1016/j.jval.2024.02.021, PMID: 38462225 PMC11176001

[46] Wateska AR Patricia Nowalk M Lin CJ Harrison LH Schaffner W Zimmerman RK. Cost-effectiveness of revised US pneumococcal vaccination recommendations in underserved minority adults < 65-years-old. Vaccine. (2022) 40:7312–20. doi: 10.1016/j.vaccine.2022.10.066, PMID: 36336526 PMC9999373

[47] United States Census Bureau. Health insurance coverage in the United States: 2019. (2020).

[48] The Commonwealth Fund. Commonwealth Fund survey of high-need patients 2016. (2016). Available online at: https://www.commonwealthfund.org/publications/surveys/2016/dec/2016-commonwealth-fund-survey-high-need-patients (Accessed March 8, 2024).

[49] The Commonwealth Fund. Medicaid cuts would affect older, sicker Americans. (2017). Available online at: https://www.commonwealthfund.org/blog/2017/medicaid-cuts-would-affect-older-sicker-americans (Accessed March 8, 2024).

[50] Burton DC Flannery B Bennett NM Farley MM Gershman K Harrison LH. Socioeconomic and racial/ethnic disparities in the incidence of bacteremic pneumonia among US adults. Am J Public Health. (2010) 100:1904–11. doi: 10.2105/AJPH.2009.181313, PMID: 20724687 PMC2936986

[51] Nowalk MP Wateska AR Lin CJ Schaffner W Harrison LH Zimmerman RK. Racial disparities in adult pneumococcal vaccination indications and pneumococcal hospitalizations in the U.S. J Natl Med Assoc. (2019) 111:540–5. doi: 10.1016/j.jnma.2019.04.011, PMID: 31171344 PMC6888932

