# Supplementary material for: Health care resource use and costs associated with adult pneumococcal disease in the United States from 2017 to 2019, stratified by age and health risk: a retrospective cohort study
Source: Front Public Health. 2025 Jul 15;13:1575125. doi: 10.3389/fpubh.2025.1575125 (PMC12303970; doi:10.3389/fpubh.2025.1575125)
Supplement: Supplementary file 1 [file Table_1.DOCX]

Supplementary Material

# Supplementary Tables

Supplementary Table 1. Pneumococcal disease codes

| Medical condition | ICD-10 codes |
| --- | --- |
| Invasive pneumococcal disease | |
| Bacteremia / septicemia | A40.3; A40.9+B95.3; A41.9+B95.3; R78.81+B95.3 |
| Meningitis | G00.1; G00.2+B95.3; G00.8/G00.9+B95.3; G03.9+B95.3 |
| Bacteremic pneumonia | J86.x+B95.3; J90/J91.8+B95.3; (A40.3, A40.9+B95.3, A41.9+B95.3, or R78.81+B95.3)+(any ACP code); (A40.9, A41.9, or R78.81)+J13 |
| Other | M00.1x; K65.8+B95.3; I30.1+B95.3; I33.0+B95.3; I33.9+B95.3; K65.2+B95.3;  M86.1x/M86.2x/M86.9+B95.3;  M00.0x/M00.2x/M00.8x/M00.9+B95.3 |
| Non-invasive pneumococcal disease | |
| Non-bacteremic pneumococcal pneumonia | 481; 482.9+041.2; 485+041.2;  486+041.2 |

ICD, International Classification of Diseases.

Supplementary Table 2. Risk group definitions

| Risk classification ^A^ | Definition |
| --- | --- |
| High risk (‘IC’) | Immunocompromising conditions (including functional or anatomic asplenia, cancer, chronic renal failure, nephrotic syndrome, HIV infection, organ transplantation), cochlear implant, or CSF leak |
| Moderate risk (‘CMC’) | Alcohol dependence, asthma, chronic heart disease (including congestive heart failure and cardiomyopathy; excluding hypertension), chronic liver disease (including hepatitis B virus infection, hepatitis C virus infection, and cirrhosis), chronic lung disease (including chronic obstructive pulmonary disease and emphysema), diabetes mellitus, or smoking |
| Low risk (‘Healthy’) | No evidence of any high- or moderate-risk condition, as listed above |

CMC, chronic medical condition; CPT, current procedural terminology; CSF, cerebrospinal fluid; HCPCS, healthcare common procedure coding system; HIV, human immunodeficiency virus; IC, immunocompromising condition; ICD, International Classification of Diseases; PCS, procedure coding system.

^A^ Categorical variable based on Advisory Committee on Immunization Practices guidelines and determined using ICD-9-CM, ICD-10-CM, ICD-9-PCS, ICD-10-PCS, and/or CPT4/HCPCS codes.

Supplementary Table 3. Cost per episode of invasive pneumococcal disease, stratified by annual cohort, age, and risk group

| Group | | Inpatient costs | | | | Outpatient costs ^A^ | | | | ED costs | | | | Total costs | | | |
| --- | --- | --- | --- | --- | --- | --- | --- | --- | --- | --- | --- | --- | --- | --- | --- | --- | --- |
|  |  | 2017 | 2018 | 2019 | 2017–19 | 2017 | 2018 | 2019 | 2017–19 | 2017 | 2018 | 2019 | 2017–19 | 2017 | 2018 | 2019 | 2017–19 |
| Overall | Mean (SD) | 43,888 (54,259) | 47,029 (60,242) | 49,266 (52,820) | 46,569 (55,933) | 2,401 (6,697) | 2,700 (5,827) | 3,359 (14,345) | 2,790 (9,457) | 88 (339) | 173 (908) | 117 (690) | 126 (682) | 46,367 (56,196) | 49,902 (61,461) | 52,742 (55,157) | 49,481 (57,734) |
|  | 95% CI | 38,117, 49,659 | 40,424, 53,633 | 43,107, 55,424 | 43,006, 50,132 | 1,689, 3,113 | 2,061, 3,339 | 1,687, 5,032 | 2,188, 3,393 | 52, 124 | 73, 272 | 37, 198 | 82, 169 | 40,390, 52,344 | 43,163, 56,640 | 46,311, 59,173 | 45,803, 53,159 |
| Age (years) |  |  |  |  |  |  |  |  |  |  |  |  |  |  |  |  |  |
| 18–49 | Mean (SD) | 39,236 (30,451) | 50,258 (73,695) | 45,946 (51,075) | 45,163 (54,505) | 2,239 (5,100) | 1,209 (1,772) | 3,210 (9,781) | 2,240 (6,555) | 87 (363) | 403 (1,561) | 177 (697) | 221 (1,007) | 41,509 (30,937) | 51,869 (73,705) | 49,333 (55,356) | 47,606 (56,004) |
|  | 95% CI | 31,629, 46,842 | 31,850, 68,666 | 33,583, 58,309 | 37,485, 52,841 | 965, 3,513 | 766, 1,651 | 843, 5,578 | 1,316, 3,163 | −3, 178 | 13, 793 | 8, 346 | 80, 363 | 33,781, 49,237 | 33,458, 70,280 | 35,934, 62,732 | 39,717, 55,496 |
| 50–64 | Mean (SD) | 50,185 (58,294) | 49,388 (61,735) | 49,688 (55,147) | 49,750 (58,555) | 1,741 (3,547) | 3,431 (6,960) | 4,211 (19,454) | 3,079 (11,544) | 70 (335) | 174 (796) | 143 (867) | 129 (699) | 51,997 (58,600) | 52,993 (63,727) | 54,043 (58,146) | 52,958 (60,258) |
|  | 95% CI | 40,996, 59,375 | 39,928, 58,849 | 40,265, 59,111 | 44,367, 55,133 | 1,182, 2,300 | 2,364, 4,498 | 887, 7,535 | 2,018, 4,140 | 17, 123 | 51, 296 | −5, 291 | 65, 193 | 42,759, 61,235 | 43,227, 62,759 | 44,107, 63,978 | 47,419, 58,498 |
| ≥65 | Mean (SD) | 38,178 (58,010) | 40,525 (45,511) | 51,303 (50,843) | 42,588 (52,507) | 3,343 (9,795) | 2,419 (5,211) | 2,106 (4,231) | 2,709 (7,258) | 112 (334) | 12 (89) | 26 (139) | 57 (235) | 41,633 (62,695) | 42,955 (46,168) | 53,435 (50,363) | 45,353 (54,656) |
|  | 95% CI | 27,737, 48,619 | 31,099, 49,950 | 40,201, 62,405 | 36,581, 48,594 | 1,580, 5,106 | 1,340, 3,498 | 1,182, 3,029 | 1,879, 3,539 | 52, 172 | −7, 30 | −5, 56 | 30, 84 | 30,348, 52,917 | 33,394, 52,517 | 42,438, 64,432 | 39,101, 51,605 |
| Health risk |  |  |  |  |  |  |  |  |  |  |  |  |  |  |  |  |  |
| Healthy | Mean (SD) | 35,184 (33,427) | 31,449 (22,948) | 39,574 (36,996) | 34,647 (30,101) | 686 (877) | 2,787 (5,660) | 2,885 (5,762) | 2,155 (4,796) | 0 (0) | 200 (688) | 244 (1,089) | 149 (706) | 35,870 (33,363) | 34,436 (24,042) | 42,702 (40,526) | 36,951 (31,519) |
|  | 95% CI | 21,386, 48,982 | 23,566, 39,332 | 22,259, 56,888 | 27,949, 41,346 | 324, 1,048 | 843, 4,731 | 188, 5,581 | 1,088, 3,222 | NA | −36, 437 | −266, 753 | −9, 306 | 22,098, 49,641 | 26,177, 42,695 | 23,735, 61,668 | 29,936, 43,965 |
| CMC | Mean (SD) | 44,179 (55,975) | 44,403 (51,961) | 57,772 (64,863) | 48,499 (57,935) | 2,041 (4,410) | 2,394 (5,429) | 4,816 (21,194) | 3,018 (12,551) | 93 (355) | 328 (1,384) | 100 (526) | 387 (168) | 46,313 (56,284) | 47,126 (53,427) | 62,688 (68,145) | 51,685 (59,741) |
|  | 95% CI | 35,023, 53,335 | 35,011, 53,796 | 46,097, 69,447 | 42,708, 54,289 | 1,320, 2,762 | 1,413, 3,375 | 1,001, 8,631 | 1,764, 4,272 | 35, 151 | 78, 579 | 5, 194 | 82, 254 | 37,107, 55,520 | 37,468, 56,783 | 50,422, 74,953 | 45,714, 57,655 |
| IC | Mean (SD) | 44,912 (55,344) | 52,180 (69,940) | 43,464 (41,476) | 46,998 (57,417) | 2,959 (8,509) | 2,902 (6,154) | 2,201 (4,914) | 2,713 (6,779) | 97 (350) | 55 (303) | 114 (114) | 88 (490) | 47,948 (58,794) | 55,138 (71,109) | 45,779 (42,194) | 49,791 (59,262) |
|  | 95% CI | 36,558, 53,267 | 41,495, 62,866 | 36,632, 50,296 | 41,859, 52,137 | 1,675, 4,244 | 1,962, 3,842 | 1,392, 3,011 | 2,106, 3,320 | 44, 150 | 9, 101 | −9, 237 | 44, 131 | 39,073, 56,824 | 44,273, 66,002 | 38,829, 52,730 | 44,487, 55,095 |
| Age/risk |  |  |  |  |  |  |  |  |  |  |  |  |  |  |  |  |  |
| 18–49, Healthy | Mean (SD) | 43,430 (41,815) | 35,585 (24,189) | 45,386 (41,961) | 40,515 (34,473) | 859 (940) | 1,759 (2,455) | 6,487 (8,065) | 2,531 (4,593) | 0 (0) | 394 (1,014) | 0 (0) | 169 (680) | 44,289 (41,636) | 37,738 (24,020) | 51,873 (49,294) | 43,215 (35,469) |
|  | 95% CI | 16,862, 69,998 | 22,190, 48,980 | 10,305, 80,466 | 28,673, 52,357 | 261, 1,456 | 399, 3,119 | −255, 13,230 | 953, 4,109 | NA | −168, 956 | NA | −65, 403 | 17,835, 70,743 | 24,436, 51,039 | 10,662, 93,084 | 30,687, 55,742 |
| 18–49, CMC | Mean (SD) | 32,523 (23,276) | 47,064 (40,782) | 59,472 (71,982) | 47,494 (52,496) | 2,451 (4,283) | 856 (1,356) | 3,078 (11,544) | 2,211 (7,601) | 148 (412) | 884 (2,540) | 218 (627) | 399 (1,484) | 35,122 (24,332) | 48,804 (41,183) | 62,768 (75,679) | 50,104 (54,739) |
|  | 95% CI | 21,928, 43,118 | 28,501, 65,628 | 30,997, 87,947 | 34,883, 60,105 | 501, 4,400 | 239, 1,474 | −1,489, 7,645 | 385, 4,037 | −39, 336 | −272, 2,040 | −30, 466 | 43, 756 | 24,046, 46,198 | 30,058, 67,551 | 32,830, 92,706 | 36,954, 63,254 |
| 18–49, IC | Mean (SD) | 42,160 (29,960) | 60,514 (104,404) | 35,016 (24,274) | 45,183 (62,050) | 2,630 (6,413) | 1,178 (1,603) | 2,524 (8,638) | 2,150 (6,401) | 80 (397) | 47 (247) | 186 (830) | 108 (562) | 44,759 (30,611) | 61,738 (104,408) | 37,725 (30,627) | 47,404 (63,029) |
|  | 95% CI | 31,170, 53,149 | 20,030, 100,997 | 26,408, 43,623 | 32,333, 58,033 | 278, 4,983 | 556, 1,800 | −539, 5,587 | 824, 3,476 | −66, 225 | −49, 142 | −108, 480 | −9, 224 | 33,531, 55,988 | 21,253, 102,223 | 26,866, 48,585 | 34,351, 60,457 |
| 50–64, Healthy | Mean (SD) | 31,105 (24,628) | 28,644 (23,364) | 36,644 (36,211) | 31,687 (27,478) | 666 (892) | 4,249 (7,902) | 502 (486) | 2,167 (5,448) | 0 (0) | 18 (72) | 443 (1,468) | 139 (801) | 31,771 (24,520) | 32,911 (26,073) | 37,589 (35,973) | 33,993 (28,264) |
|  | 95% CI | 13,487, 48,723 | 16,194, 41,093 | 12,318, 60,971 | 22,526, 40,849 | 28, 1,304 | 38, 8,460 | 176, 829 | 350, 3,983 | NA | −20, 56 | −544, 1,429 | −128, 406 | 14,230, 49,312 | 19,017, 46,804 | 13,422, 61,756 | 24,570, 43,417 |
| 50–64, CMC | Mean (SD) | 51,262 (65,646) | 47,712 (59,026) | 59,402 (70,252) | 52,516 (64,892) | 1,599 (3,250) | 3,147 (6,890) | 7,240 (30,068) | 3,712 (16,810) | 61 (348) | 354 (1,233) | 84 (624) | 159 (802) | 52,922 (66,097) | 51,212 (61,527) | 66,726 (75,221) | 56,387 (67,500) |
|  | 95% CI | 36,262, 66,263 | 32,330, 63,094 | 40,410, 78,394 | 43,230, 61,803 | 856, 2,341 | 1,351, 4,942 | −889, 15,369 | 1,307, 6,118 | −19, 140 | 32, 675 | −84, 253 | 44, 273 | 37,818, 68,026 | 35,178, 67,246 | 46,391, 87,061 | 46,727, 66,047 |
| 50–64, IC | Mean (SD) | 51,720 (53,126) | 54,122 (67,453) | 43,942 (41,528) | 50,371 (56,337) | 2,045 (4,043) | 3,472 (6,902) | 2,361 (3,047) | 2,703 (5,181) | 90 (345) | 84 (385) | 142 (917) | 103 (584) | 53,855 (53,215) | 57,678 (69,258) | 46,445 (41,507) | 53,177 (57,241) |
|  | 95% CI | 39,145, 64,295 | 40,074, 68,170 | 33,890, 53,994 | 43,051, 57,690 | 1,088, 3,002 | 2,034, 4,909 | 1,624, 3,099 | 2,030, 3,376 | 8, 172 | 4, 164 | −80, 364 | 27, 179 | 41,259, 66,451 | 43,254, 72,101 | 36,398, 56,492 | 45,740, 60,614 |
| ≥65, Healthy | Mean (SD) | 15,798 (2,718) | 27,157 (18,881) | 25,301 (NA) | 22,666 (13,697) | 59 (102) | 796 (849) | 269 (NA) | 454 (671) | 0 (0) | 204 (407) | 0 (0) | 102 (288) | 15,858 (2,629) | 28,157 (18,435) | 25,570 (NA) | 23,221 (13,623) |
|  | 95% CI | 9,047, 22,550 | −2,886, 57,200 | NA | 11,214, 34,117 | −195, 314 | −555, 2,148 | NA | −107, 1,015 | NA | −444, 851 | NA | −139, 342 | 9,327, 22,389 | −1,178, 57,492 | NA | 11,832, 34,610 |
| ≥65, CMC | Mean (SD) | 38,188 (48,271) | 38,125 (46,294) | 54,297 (52,048) | 43,077 (49,040) | 2,551 (5,827) | 2,091 (4,020) | 2,600 (5,183) | 2,422 (5,087) | 119 (342) | 0 (0) | 40 (196) | 58 (242) | 40,858 (48,397) | 40,216 (46,198) | 56,936 (51,457) | 45,556 (48,890) |
|  | 95% CI | 24,323, 52,053 | 23,320, 52,931 | 37,425, 71,169 | 34,499, 51,654 | 877, 4,225 | 805, 3,377 | 920, 4,280 | 1,532, 3,312 | 21, 218 | NA | −24, 103 | 15, 100 | 26,957, 54,760 | 25,441, 54,991 | 40,256, 73,616 | 37,005, 54,107 |
| ≥65, IC | Mean (SD) | 39,144 (65,349) | 43,638 (46,682) | 49,193 (50,666) | 43,193 (56,263) | 4,048 (11,996) | 2,828 (6,216) | 1,700 (3,189) | 3,051 (8,752) | 112 (337) | 5 (36) | 14 (53) | 54 (228) | 43,303 (72,459) | 46,471 (47,923) | 50,907 (50,200) | 46,297 (59,996) |
|  | 95% CI | 23,445, 54,842 | 30,083, 57,193 | 33,600, 64,786 | 34,408, 51,977 | 1,166, 6,929 | 1,023, 4,633 | 719, 2,681 | 1,684, 4,417 | 31, 193 | −5, 16 | −2, 30 | 18, 89 | 25,897, 60,710 | 32,556, 60,386 | 35,458 66,356 | 36,929, 55,665 |

CI, confidence interval; CMC, chronic medical condition; ED, emergency department; IC, immunocompromising condition; NA, not applicable; SD, standard deviation.

All values are expressed in US dollars. All individuals with invasive pneumococcal disease were hospitalized.

^A^ Includes all pneumococcal disease-related visits and procedures in ambulatory settings. Does not include pharmacy costs.

Supplementary Table 4. Cost per episode of non-bacteremic pneumococcal pneumonia with hospitalization, stratified by annual cohort, age, and risk group

| Group | | Inpatient costs | | | | Outpatient costs ^A^ | | | | ED costs | | | | Total costs | | | |
| --- | --- | --- | --- | --- | --- | --- | --- | --- | --- | --- | --- | --- | --- | --- | --- | --- | --- |
|  |  | 2017 | 2018 | 2019 | 2017–19 | 2017 | 2018 | 2019 | 2017–19 | 2017 | 2018 | 2019 | 2017–19 | 2017 | 2018 | 2019 | 2017–19 |
| Overall | Mean (SD) | 21,307 (28,968) | 24,010 (26,477) | 30,574 (41,766) | 24,858 (32,731) | 1,948 (6,544) | 2,148 (8,264) | 2,894 (18,166) | 2,287 (11,588) | 121 (476) | 224 (1,155) | 230 (986) | 185 (886) | 23,376 (31,660) | 26,382 (28,724) | 33,698 (45,801) | 27,330 (35,786) |
|  | 95% CI | 16,770, 25,845 | 19,284, 28,736 | 22,926, 38,221 | 21,636, 28,079 | 923, 2,973 | 673, 3,623 | −432, 6,221 | 1,147, 3,428 | 47, 196 | 18, 430 | 50, 411 | 98, 272 | 18,417, 28,335 | 21,255, 31,509 | 25,312, 42,085 | 23,807, 30,852 |
| Age (years) |  |  |  |  |  |  |  |  |  |  |  |  |  |  |  |  |  |
| 18–49 | Mean (SD) | 21,051 (18,588) | 30,541 (27,737) | 17,869 (11,473) | 23,516 (21,283) | 773 (961) | 634 (1,044) | 1,421 (2,433) | 910 (1,567) | 154 (435) | 134 (539) | 638 (1,320) | 286 (838) | 21,978 (18,850) | 31,309 (28,439) | 19,927 (11,526) | 24,711 (21,591) |
|  | 95% CI | 13,543, 28,559 | 19,338, 41,744 | 12,647, 23,091 | 18,550, 28,481 | 384, 1,161 | 212, 1,056 | 314, 2,529 | 544, 1,275 | −22, 330 | −84, 352 | 37, 1,238 | 91, 481 | 14,364, 29,591 | 19,822, 42,795 | 14,681, 25,174 | 19,674, 29,749 |
| 50–64 | Mean (SD) | 23,166 (36,597) | 24,239 (21,357) | 32,336 (50,665) | 26,651 (38,944) | 616 (1,303) | 3,356 (10,509) | 4,597 (25,484) | 2,771 (16,038) | 143 (656) | 448 (1,778) | 220 (1,120) | 256 (1,218) | 23,925 (36,828) | 28,043 (26,284) | 37,153 (56,869) | 29,678 (42,804) |
|  | 95% CI | 13,950, 32,383 | 18,037, 30,440 | 19,132, 45,539 | 20,755, 32,548 | 288, 944 | 304, 6,407 | −2,044, 11,239 | 343, 5,200 | −23, 308 | −68, 964 | −72, 512 | 71, 440 | 14,650, 33,200 | 20,410, 35,675 | 22,333, 51,973 | 23,198, 36,159 |
| ≥65 | Mean (SD) | 19,729 (24,230) | 20,321 (29,957) | 34,974 (35,958) | 23,531 (29,675) | 3,583 (9,557) | 1,768 (7,876) | 1,015 (2,004) | 2,404 (7,875) | 90 (247) | 51 (235) | 16 (55) | 61 (214) | 23,402 (30,769) | 22,140 (31,065) | 36,005 (36,599) | 25,995 (32,605) |
|  | 95% CI | 13,951, 25,506 | 11,716, 28,925 | 22,986, 46,963 | 18,837, 28,224 | 1,305, 5,862 | −494, 4,030 | 347, 1,683 | 1,158, 3,650 | 32, 149 | −16, 119 | −2, 34 | 27, 94 | 16,066, 30,739 | 13,217, 31,063 | 23,802, 48,208 | 20,838, 31,152 |
| Health risk |  |  |  |  |  |  |  |  |  |  |  |  |  |  |  |  |  |
| Healthy | Mean (SD) | 23,980 (21,142) | 28,908 (34,291) | 31,210 (36,841) | 27,005 (28,203) | 646 (999) | 557 (525) | 2,747 (3,719) | 1,053 (1,947) | 73 (274) | 386 (894) | 0 (0) | 155 (537) | 24,699 (21,647) | 29,852 (35,440) | 33,956 (38,251) | 28,213 (29,186) |
|  | 95% CI | 11,773, 36,187 | 2,549, 55,266 | −7,453, 69,872 | 16,277, 37,733 | 69, 1,223 | 154, 961 | −1,156, 6,650 | 313, 1,793 | −85, 232 | −301, 1,073 | NA | −49, 360 | 12,200, 37,198 | 2,610, 57,093 | −6,185, 80,283 | 17,112, 39,315 |
| CMC | Mean (SD) | 21,167 (35,035) | 22,498 (28,911) | 35,623 (56,042) | 25,794 (41,085) | 1,762 (4,405) | 2,039 (7,673) | 5,052 (27,130) | 2,807 (15,479) | 122 (436) | 412 (1,683) | 355 (1,069) | 278 (1,127) | 23,051 (35,441) | 24,950 (30,094) | 41,031 (62,109) | 28,879 (44,141) |
|  | 95% CI | 12,934, 29,399 | 14,607, 30,389 | 20,021, 51,226 | 19,717, 31,871 | 727, 2,797 | −55, 4,133 | −2,501, 12,605 | 518, 5,097 | 20, 225 | −47, 872 | 57, 653 | 111, 445 | 14,722, 31,379 | 16,736, 33,164 | 23,740, 58,322 | 22,350, 35,409 |
| IC | Mean (SD) | 20,933 (23,426) | 24,636 (23,090) | 26,058 (23,802) | 23,665 (23,420) | 2,381 (8,608) | 2,485 (9,369) | 1,007 (1,944) | 1,991 (7,525) | 130 (543) | 29 (170) | 144 (956) | 103 (632) | 23,444 (29,595) | 27,150 (26,793) | 27,209 (24,214) | 25,759 (27,079) |
|  | 95% CI | 15,468, 26,399 | 18,671, 30,601 | 19,855, 32,261 | 20,331, 26,999 | 373, 4,389 | 64, 4,905 | 501, 1,514 | 920, 3,062 | 4, 257 | −15, 73 | −105, 393 | 13, 193 | 16,539, 30,349 | 20,229, 34,071 | 20,899, 33,520 | 21,905, 29,614 |
| Age/risk |  |  |  |  |  |  |  |  |  |  |  |  |  |  |  |  |  |
| 18–49, Healthy | Mean (SD) | 35,615 (29,503) | 34,901 (42,520) | 14,215 (6,745) | 28,811 (29,261) | 1,126 (1,295) | 659 (826) | 2,445 (4,045) | 1,388 (2,324) | 205 (459) | 797 (1,305) | 0 (0) | 324 (782) | 36,946 (29,576) | 36,357 (44,645) | 16,660 (3,223) | 30,523 (29,750) |
|  | 95% CI | −1,019, 72,248 | −32,757, 102,560 | 3,481, 24,948 | 11,129, 46,493 | −482, 2,734 | −656, 1,974 | −3,991, 8,882 | −16, 2,792 | −365, 775 | −1,279, 2,874 | NA | −148, 797 | 223, 73,669 | −34,683, 107,397 | 11,532, 21,788 | 12,545, 48,501 |
| 18–49, CMC | Mean (SD) | 13,320 (7,074) | 31,285 (28,994) | 18,442 (13,963) | 22,555 (21,392) | 451 (307) | 426 (559) | 784 (996) | 575 (736) | 451 (776) | 0 (0) | 1,116 (1,608) | 537 (1,159) | 14,223 (7,268) | 31,712 (29,186) | 20,342 (14,088) | 23,666 (21,385) |
|  | 95% CI | 5,896, 20,744 | 12,863, 49,707 | 9,571, 27,314 | 14,567, 30,543 | 129, 773 | 71, 782 | 152, 1,417 | 300, 849 | −363, 1,266 | NA | 94, 2,138 | 104, 970 | 6,596, 21,849 | 13,168, 50,256 | 11,391, 29,293 | 15,681, 31,651 |
| 18–49, IC | Mean (SD) | 19,290 (15,676) | 27,903 (22,136) | 19,416 (8,397) | 22,182 (17,244) | 783, 1,024 | 873 (1,516) | 2,130 (3,422) | 1,038 (1,756) | 18 (69) | 30 (94) | 0 (0) | 19 (71) | 20,091 (16,003) | 28,806 (22,859) | 21,547 (9,812) | 23,238 (17,759) |
|  | 95% CI | 10,608, 27,971 | 12,068, 43,738 | 8,990, 29,843 | 15,743, 28,621 | 216, 1,350 | −211, 1,958 | −2,119, 6,379 | 382, 1,693 | −20, 56 | −38, 97 | NA | −8, 45 | 11,228, 28,953 | 12,453, 45,159 | 9,363, 33,730 | 16,607, 29,870 |
| 50–64, Healthy | Mean (SD) | 13,409 (2,963) | 27,917 (33,839) | NER | 18,245 (19,207) | 134 (149) | 524(112) | NER | 264 (233) | 0 (0) | 72 (144) | NER | 24 (83) | 13,542 (2,988) | 28,513 (33,984) | NER | 18,532 (19,365) |
|  | 95% CI | 10,932, 15,886 | −25,929, 81,763 | NA | 6,041, 30,448 | 9, 258 | 346, 702 | NA | 116, 412 | NA | −157, 301 | NA | −29, 77 | 11,044, 16,040 | −25,564, 82,590 | NA | 6,229, 30,836 |
| 50–64, CMC | Mean (SD) | 28,452 (50,109) | 16,008 (13,536) | 39,634 (71,595) | 29,123 (53,232) | 826 (1,661) | 1,386 (3,067) | 9,011 (37,536) | 3,765 (22,081) | 109 (526) | 1,061 (2,672) | 181 (890) | 374 (1,509) | 29,386 (50,345) | 18,455 (15,765) | 48,826 (80,142) | 33,263 (57,923) |
|  | 95% CI | 10,386, 46,518 | 9,673, 22,343 | 11,312, 67,956 | 17,200, 41,047 | 227, 1,425 | −49, 2,822 | −5,837, 23,860 | −1,181, 8,711 | −81, 299 | −190, 2,311 | −171, 533 | 37, 712 | 11,235, 47,538 | 11,077, 25,834 | 17,122, 80,529 | 20,289, 46,237 |
| 50–64, IC | Mean (SD) | 19,207 (11,920) | 30,484 (23,008) | 26,178 (20,448) | 25,457 (19,541) | 492 (853) | 5,469 (14,438) | 873 (1,459) | 2,158 (8,209) | 239 (897) | 0 (0) | 253 (1,296) | 172 (953) | 19,937 (12,196) | 35,954 (30,175) | 27,304 (21,098) | 27,787 (22,938) |
|  | 95% CI | 14,052, 24,362 | 20,769, 40,200 | 18,806, 33,550 | 21,080, 29,834 | 123, 860 | −628, 11,566 | 347, 1,399 | 319, 3,997 | −149, 627 | NA | −214, 720 | −41, 386 | 14,663, 25,211 | 23,212, 48,696 | 19,698, 34,911 | 22,649, 32,925 |
| ≥65, Healthy | Mean (SD) | 50,373 (NA) | 8,899 (NA) | 65,200 (56,426) | 47,418 (42,066) | 2,341 (NA) | 284 (NA) | 3,350 (4,356) | 2,331 (2,901) | 0 (0) | 0 (0) | 0 (0) | 0 (0) | 52,714 (NA) | 9,182 (NA) | 68,549 (60,782) | 49,749 (44,929) |
|  | 95% CI | NA | NA | −441,766, 572,165 | −19,519, 114,355 | NA | NA | −35,790, 42,489 | −2,285, 6,947 | NA | NA | NA | NA | NA | NA | −477,555, 614,653 | −21,743, 121,240 |
| ≥65, CMC | Mean (SD) | 15,694 (13,458) | 23,605 (37,654) | 43,154 (40,873) | 23,390 (30,412) | 2,875 (6,058) | 3,512 (11,651) | 770 (1,096) | 2,682 (7,803) | 76 (189) | 48 (224) | 16 (57) | 56 (184) | 18,645 (15,504) | 27,165 (39,284) | 43,940 (41,504) | 26,127 (31,404) |
|  | 95% CI | 10,999, 20,390 | 6,911, 40,300 | 18,455, 67,853 | 16,085, 30,696 | 761, 4,989 | −1,653, 8,678 | 107, 1,432 | 807, 4,556 | 10, 142 | −52, 147 | −19, 50 | 11.4 - 100.0 | 13,236, 24,055 | 9,748, 44,583 | 18,859, 69,020 | 18,583, 33,672 |
| ≥65, IC | Mean (SD) | 22,772 (31,021) | 17,981 (22,620) | 27,393 (30,358) | 22,496 (28,354) | 4,307 (12,192) | 349 (473) | 947 (2,175) | 2,177 (8,143) | 107 (296) | 56 (253) | 18 (57) | 67 (241) | 27,186 (40,316) | 18,387 (22,650) | 28,358 (30,577) | 24,740 (32,995) |
|  | 95% CI | 12,116, 33,428 | 8,844, 27,117 | 13,933, 40,853 | 16,305, 28,687 | 119, 8,495 | 158, 540 | −17, 1,912 | 399, 3,955 | 5, 209 | −46, 159 | −8, 43 | 14.7 - 120.0 | 13,337, 41,035 | 9,238, 27,535 | 14,801, 41,915 | 17,536, 31,945 |

CI, confidence interval; CMC, chronic medical condition; ED, emergency department; IC, immunocompromising condition; NA, not applicable; NER, no episodes recorded; SD, standard deviation.

All values are expressed in US dollars.

^A^ Includes all pneumococcal disease-related visits and procedures in ambulatory settings. Does not include pharmacy costs.

Supplementary Table 5. Cost per episode of non-bacteremic pneumococcal pneumonia with no hospitalization, stratified by annual cohort, age, and risk group

| Group | | Outpatient costs ^A^ | | | | ED costs | | | | Total costs | | | |
| --- | --- | --- | --- | --- | --- | --- | --- | --- | --- | --- | --- | --- | --- |
|  |  | 2017 | 2018 | 2019 | 2017–19 | 2017 | 2018 | 2019 | 2017–19 | 2017 | 2018 | 2019 | 2017–19 |
| Overall | Mean (SD) | 788 (2,422) | 929 (3,017) | 736 (2,567) | 817 (2,648) | 189 (1,311) | 212 (1,705) | 495 (4,268) | 273 (2,493) | 977 (2,774) | 1,141 (3,550) | 1,231 (4,998) | 1,090 (3,672) |
|  | 95% CI | 629, 948 | 685, 1,174 | 509, 964 | 700, 935 | 102, 275 | 74, 350 | 117, 873 | 162, 383 | 794, 1,160 | 853, 1,429 | 789, 1,674 | 927, 1,252 |
| Age (years) |  |  |  |  |  |  |  |  |  |  |  |  |  |
| 18–49 | Mean (SD) | 413 (1,178) | 404 (894) | 566 (1,718) | 452 (1,277) | 179 (902) | 231 (1,059) | 573 (4,082) | 304 (2,294) | 592 (1,593) | 635 (1,439) | 1,139 (4,415) | 756 (2,658) |
|  | 95% CI | 254, 571 | 266, 542 | 282, 850 | 342, 562 | 58, 301 | 68, 394 | –102, 1,248 | 106, 501 | 377, 807 | 413, 857 | 409, 1,869 | 527, 985 |
| 50–64 | Mean (SD) | 531 (1,367) | 885 (3,132) | 518 (1,004) | 637 (2,026) | 178 (1,371) | 179 (1,541) | 648 (5,374) | 308 (3,084) | 709 (1,991) | 1,063 (3,647) | 1,166 (5,542) | 945 (3,774) |
|  | 95% CI | 385, 677 | 497, 1,272 | 386, 649 | 497, 776 | 32, 325 | –12, 370 | –56, 1,353 | 97, 520 | 496, 922 | 612, 1,515 | 439, 1,892 | 686, 1,204 |
| ≥65 | Mean (SD) | 1,294 (3,528) | 1,506 (3,950) | 1,336 (4,555) | 1,359 (3,859) | 206 (1,466) | 243 (2,340) | 123 (668) | 200 (1,645) | 1,499 (3,798) | 1,749 (4,615) | 1,459 (4,595) | 1,559 (4,190) |
|  | 95% CI | 913, 1,675 | 906, 2,106 | 522, 2,149 | 1,056, 1,663 | 47, 364 | –112, 599 | 4, 242 | 70, 329 | 1,089, 1,909 | 1,048, 2,450 | 639, 2,279 | 1,230, 1,888 |
| Health risk |  |  |  |  |  |  |  |  |  |  |  |  |  |
| Healthy | Mean (SD) | 248 (432) | 381 (925) | 368 (803) | 323 (722) | 16 (153) | 175 (1,077) | 143 (928) | 101 (780) | 264 (488) | 557 (1,641) | 511 (1,209) | 423 (1,157) |
|  | 95% CI | 180, 315 | 212, 551 | 219, 518 | 251, 395 | –8, 40 | –22, 373 | –30, 316 | 23, 178 | 187, 340 | 256, 857 | 285, 736 | 308, 538 |
| CMC | Mean (SD) | 744 (2,307) | 654 (1,665) | 745 (2,816) | 719 (2,280) | 184 (1,248) | 347 (2,394) | 533 (3,479) | 311 (2,274) | 928 (2,635) | 1,001 (2,947) | 1,278 (4,483) | 1,029 (3,235) |
|  | 95% CI | 533, 955 | 453, 854 | 370, 1,120 | 573, 864 | 70, 298 | 59, 636 | 70, 996 | 166, 456 | 687, 1,169 | 646, 1,356 | 681, 1,875 | 823, 1,235 |
| IC | Mean (SD) | 1,196 (3,156) | 1,611 (4,647) | 984 (2,979) | 1,276 (3,669) | 302 (1,737) | 55 (405) | 693 (6,238) | 322 (3,361) | 1,498 (3,607) | 1,666 (4,810) | 1,676 (6,926) | 1,598 (5,014) |
|  | 95% CI | 813, 1,579 | 966, 2,256 | 519, 1,449 | 987, 1,564 | 91, 513 | –1, 111 | –281, 1,667 | 58, 586 | 1,060, 1,936 | 998, 2,333 | 595, 2,758 | 1,204, 1,992 |
| Age/risk |  |  |  |  |  |  |  |  |  |  |  |  |  |
| 18–49, Healthy | Mean (SD) | 195 (147) | 376 (997) | 431 (1,099) | 320 (818) | 23 (201) | 156 (1,082) | 6 (45) | 60 (622) | 218 (258) | 532 (1,544) | 437 (1,098) | 380 (1,063) |
|  | 95% CI | 162, 228 | 121, 631 | 134, 728 | 203, 436 | –23, 69 | –121, 433 | –6, 18 | –28, 148 | 159, 277 | 136, 927 | 141, 734 | 229, 531 |
| 18–49, CMC | Mean (SD) | 373 (1,116) | 451 (962) | 621 (2,255) | 459 (1,448) | 125 (678) | 374 (1,227) | 1,218 (6,215) | 477 (3,230) | 499 (1,279) | 825 (1,584) | 1,838 (6,592) | 936 (3,543) |
|  | 95% CI | 155, 591 | 227, 675 | 33, 1,208 | 273, 646 | –7, 258 | 88, 661 | –402, 2,837 | 61.8 - 892.1 | 249, 748 | 456, 1,195 | 120, 3,556 | 481, 1,392 |
| 18–49, IC | Mean (SD) | 1,026 (2,135) | 348 (361) | 710 (1,429) | 708 (1,542) | 696 (1,843) | 35 (192) | 338 (1,707) | 371 (1,481) | 1,722 (3,083) | 383 (537) | 1,048 (2,120) | 1,080 (2,276) |
|  | 95% CI | 281, 1,770 | 213, 482 | 166, 1,253 | 391, 1,026 | 53, 1,339 | –37, 107 | –311, 988 | 66, 676 | 646, 2,798 | 182, 583 | 242, 1,854 | 611, 1,548 |
| 50–64, Healthy | Mean (SD) | 257 (381) | 383 (890) | 306 (342) | 308 (565) | 12 (100) | 155 (1,060) | 303 (1,357) | 145 (952) | 269 (472) | 539 (1,815) | 610 (1,380) | 453 (1,276) |
|  | 95% CI | 163, 350 | 122, 645 | 211, 402 | 222, 395 | –12, 37 | –156, 467 | –74, 681 | –2, 291 | 153, 385 | 6, 1,072 | 225, 994 | 257, 649 |
| 50–64, CMC | Mean (SD) | 508 (1,426) | 489 (768) | 445 (885) | 487 (1,142) | 191 (1,580) | 292 (2,133) | 393 (1,857) | 270 (1,821) | 698 (2,189) | 781 (2,362) | 838 (2,222) | 757 (2,244) |
|  | 95% CI | 304, 712 | 350, 628 | 270, 621 | 376, 598 | –35, 417 | –95, 680 | 25, 762 | 93, 447 | 385, 1,012 | 353, 1,210 | 397, 1,279 | 539, 975 |
| 50–64, IC | Mean (SD) | 801 (1,661) | 1,697 (5,147) | 764 (1,364) | 1,109 (3,325) | 282 (1,400) | 36 (281) | 1,235 (9,085) | 483 (5,074) | 1,083 (2,195) | 1,732 (5,371) | 1,999 (9,251) | 1,592 (6,139) |
|  | 95% CI | 438, 1,164 | 600, 2,794 | 448, 1,080 | 690, 1,528 | –23, 588 | –24, 96 | –870, 3,340 | –156, 1,123 | 604, 1,562 | 588, 2,877 | –144, 4,142 | 818, 2,367 |
| ≥65, Healthy | Mean (SD) | 450 (1,049) | 405 (627) | 330 (346) | 415 (832) | 0 (0) | 410 (1,231) | 0 (0) | 115 (653) | 450 (1,049) | 816 (1,457) | 330 (346) | 530 (1,082) |
|  | 95% CI | –90, 989 | –77, 888 | –34, 694 | 115, 715 | NA | –536, 1,357 | NA | –120, 351 | –90, 989 | –304, 1,935 | –34, 694 | 140, 920 |
| ≥65, CMC | Mean (SD) | 1,235 (3,343) | 1,113 (2,797) | 1,367 (4,735) | 1,231 (3,533) | 212 (1,089) | 408 (3,445) | 92 (494) | 237 (1,901) | 1,447 (3,497) | 1,520 (4,416) | 1,459 (4,753) | 1,468 (3,993) |
|  | 95% CI | 728, 1,743 | 469, 1,756 | 144, 2,590 | 832, 1,630 | 47, 378 | –385, 1,200 | –36, 220 | 22, 451 | 916, 1,978 | 504, 2,537 | 231, 2,687 | 1,017, 1,918 |
| ≥65, IC | Mean (SD) | 1,460 (3,903) | 1,969 (4,878) | 1,408 (4,633) | 1,600 (4,348) | 222 (1,878) | 81 (545) | 169 (843) | 170 (1,418) | 1,682 (4,298) | 2,050 (4,999) | 1,577 (4,693) | 1,770 (4,580) |
|  | 95% CI | 821, 2,098 | 917, 3,021 | 179, 2,637 | 1,096, 2,104 | –85, 529 | –37, 198 | –55, 393 | 5, 334 | 979, 2,385 | 972, 3,128 | 332, 2,822 | 1,239, 2,301 |

CI, confidence interval; CMC, chronic medical condition; ED, emergency department; IC, immunocompromising condition; NA, not applicable; NER, no episodes recorded; SD, standard deviation.

All values are expressed in US dollars.

^A^ Includes all pneumococcal disease-related visits and procedures in ambulatory settings. Does not include pharmacy costs.
